# Antecedents and outcomes of cyberbullying among Chinese university students: verification of a behavioral pathway model

**DOI:** 10.3389/fpubh.2024.1359828

**Published:** 2024-04-02

**Authors:** Jian-Hong Ye, Xiantong Yang, Weiguaju Nong, Mengqin Wang, Yi-Sang Lee

**Affiliations:** ^1^Faculty of Education, Beijing Normal University, Beijing, China; ^2^National Institute of Vocational Education, Beijing Normal University, Beijing, China; ^3^Faculty of Psychology, Beijing Normal University, Beijing, China; ^4^School of Education, Guangxi University of Foreign Languages, Nanning, China; ^5^Department of Lifelong Learning, Simon Fraser University, Vancouver, BC, Canada; ^6^Department of Industrial Education, National Taiwan Normal University, Taipei City, Taiwan

**Keywords:** cognitive-behaviors-results model, continued cyberbullying intention, excessive moral sense, happiness, overconfidence, perceived value, self-perception, too far is as bad as not enough

## Abstract

**Introduction:**

Cyberbullying is a commonly-seen and hotly-debated social topic around the globe. This negative behavior is the source of many disastrous events, and so leading government bodies, organizations, schools and social communities attach great importance to addressing this topic. However, there is still much work to do in order to be clear about the causes of cyberbullying.

**Methods:**

The previous research cases were mostly viewed from the victims’ perspectives; however, there is no comprehensive understanding of the perpetrators’ viewpoints. Therefore, based on Social Cognitive Theory (SCT) and analysis of discussion in the literature, the following six variables were chosen as the focus of this study: overconfidence, excessive moral sense, cyberbullying, perceived value, happiness, and continued cyberbullying intention. This study established a research model of continued cyberbullying intention, which was verified by Structural Equation Modeling. In order to achieve the aims of the study, Chinese university students with an average age of 20.29 (*SD* = 1.43) were recruited as participants, from whom 1,048 valid questionnaires were collected.

**Results:**

The research results are as follows: 1. Overconfidence and excessive moral sense positively predicted cyberbullying behaviors; 2. Overconfidence positively predicted excessive moral sense; 3. Cyberbullying positively predicted perceived value and sense of happiness; and 4. Perceived value and sense of happiness positively predicted continued cyberbullying intentions.

**Conclusion:**

Students’ biased self-perception significantly predicts their cyberbullying behaviors and continued cyberbullying intention. What is more, it is interesting to learn that perpetrators will continue to exhibit cyberbullying behaviors when they believe that what they do (cyberbullying) is valuable or allows them to experience positive feelings; this requires our attention.

## 1 Introduction

Bullying is a topic which requires considerable attention. Although people know that it is not moral and should not be done, this type of behavior still continues to occur. From TV dramas, films, news reports, and other life events, we can see that this issue should not be ignored by the general public. In this era, traditional, in-person bullying has developed into various forms and has emerged through various different pathways. What is more, the perpetrators of cyberbullying remain unknown, which results in cyber attacking, cyber info leaks (Wangluocesuo, 网络厕所), cyber doxing, online celebrity fans culture (Fanquanwenhua, 饭圈文化), and so on. In recent years, it has evolved into a global public health issue. Therefore, it is necessary to explore the formative factors and results of this phenomenon. The results will be helpful for parents, teachers, schools, and government bodies to develop better preventative education and intervention strategies.

In this digital era, technological development allows people to visit websites and have access to information from around the globe. However, this has also led to the transborder nature of cyberbullying (1, 2). Cyberbullying has thus become a social issue which can be commonly witnessed all over the world. In consideration of the negative influence of cyberbullying on its victims, a growing number of studies have started to discover the factors related to cyberbullying (3). Cyberbullying is considered to have effects on people in any age group; as long as they can use technology, cyberbullying can occur through text messages, emails, online chatting, social media and other multiple ways (4). The influence can be aggravated as time goes by, and can eventually harm one’s mental health (5). Accordingly, cyberbullying is a non-physical, malicious attack by perpetrators (keyboard warriors) against victims, based on Internet technology. This method can be either public or non-public, either anonymous or using real names, either individual or in groups. It involves sending malicious texts, audios, or videos to victims, and could be random or non-random targeted behavior. Although there is no direct physical harm to victims, it still creates a severe burden and has an extremely negative influence on their psychological and mental wellbeing. Therefore, the severity of cyberbullying leads to the consistent emergence of many unfortunate events. Considering the high popularity and the negative influence of cyberbullying on victims, it is necessary to examine the related factors (3). In order to build a useful and consistent knowledge system, it is important to reach a certain degree of consensus on defining the phenomenon in terms of scientific concepts; efforts to measure cyberbullying are therefore conducted “in the context of bullying” (6). This article thus focuses on cyberbullying with the aim of stimulating further research.

Santre (7) believes that research on cyberbullying is necessary, based on the increasing use of the Internet and the negative influence of cyberbullying on teenagers. The research on cyberbullying has a history of less than 20 years, but has experienced exponential growth in recent years (8). For example, there are research cases exploring the relation between intervention (zero intervention) by parents and teachers and the bullying behavior of students (9); the use of social media and students’ cyberbullying behaviors (10); battered children and the influence of cyberbullying behaviors on university students (11); prediction of cyberbullying behavior by teenagers (12); bullied experiences during high school, and adaptation and motivation in university (13); and the predictive factors of cyberbullying at university and its relativity with personality traits (14). The bullying issue has been emphasized by advocates including education workers, counselors, researchers, and policy makers (15). Although there are already many researchers focusing on this area, these events still occur frequently; therefore, there is still a necessity for further research in this area. That is because, as some studies have found, cyberbullying can not only directly lead to some teenagers having behaviors which are harmful to their own health, but it is also a possible cause of depression. More severely, some of those involved may even have extreme behaviors and commit suicide (15, 16). That is to say, cyberbullying is a commonly-seen public health issue which has negative influence on teenagers. Furthermore, the rising occurrence of cyberbullying comes from the increasing use of electronic devices and the rising popularity of the Internet.

The previous research has pointed out that it is of great value to measure how young people view the various factors related to cyberbullying (17). Cyberbullying is a socio-psychological development caused by mediation, and this is a meta-process which shapes daily practices and social relations, based on technology and media (8). Therefore, it is considered that Social Cognitive Theory (SCT) can be used to interpret bullying behaviors. From a cognitive perspective, teenagers are not likely to bully others when they believe these behaviors are unacceptable. This is the area in which cognition and reinforcement play critical roles. In other words, the feelings of participants (perpetrators) can encourage or discourage the behaviors of the leading target or the target in order to avoid harm (18). Therefore, when people have cognitive bias, they are more likely to demonstrate negative behaviors. To be specific, when people believe that they are right, or they belong to the righteous party, they are more likely to blame others. Of course, this type of excessive moral sense could lead to cyberbullying. This is in accordance with the perspective of Veenstra et al. (18) that the perpetrators mainly aim at controlling others.

Although researchers have made significant progress, there are still gaps to be filled in terms of knowledge of cyberbullying (19). In the real world, according to media reports, the perpetrators are considerably confident and believe that they have a moral sense. They do not realize that they are bullying others. While this issue has attracted public attention, it has rarely become a research topic. Therefore, this research aimed to start from the wrong cognition, to discover the factors leading to continued cyberbullying intentions. This is a new and less popular perspective, which will help us better understand the theory and pathway of cyberbullying behaviors.

### 1.1 Overconfidence

According to research, the judgment and decisions of people are influenced by a series of cognitive, perceptive and motivational biases. However, people are not always precise and objective in their perceptions of themselves, their environments and the surrounding people (20). Therefore, misperceptions and over-cognition frequently occur. This type of over-certainty of people’s own beliefs, or over-accuracy, is called over-confidence (21); it is the most consistent, powerful, and common cognitive bias (22) which universally exists in indirect interpersonal social relations (23). An unreasonable cognitive bias leads to negative consequences, for example, believing that only oneself is right. Therefore, this research took overconfidence as an independent variable, and further examined its influence on cyberbullying behavior. In this research, overconfidence is defined as the inconsistency between people’s perceived self-confidence (which is higher) and the real situation. Humans have many types of cognitive bias, among which one of the most consistent, powerful and common biases is overconfidence (22). Overconfidence means that one is over-certain about one’s own beliefs (21). What is more, overconfidence is a high cognitive bias which universally exists in indirect interpersonal social relations (23). Negative cognitive bias also leads to some negative consequences such as believing that only oneself is right. Therefore, this research took overconfidence as one of the independent variables in order to examine its influence on the behavioral pathway of cyberbullying. In this research, overconfidence is defined as the inconsistency between a person’s perceived self-confidence (which is higher) and the real situation.

### 1.2 Excessive moral sense

It is certain that there are various types of cognitive biases; another commonly seen bias is called excessive moral sense, which can also be called hypocrisy. Interpreting excessive moral sense as hypocrisy, those who have an excessive moral sense not only support some criteria, but also believe that it is, to some extent, the reflection of their own moral standard (24). What is more, according to Zhou (25, 26), excessive moral sense has various forms; they either emphasize that a certain value should be an absolute moral standard, or they start from individual emotions and exaggerate the role of moral judgment. Therefore, the comments and behaviors from the supporters of an excessive moral sense are very likely to place a burden on and cause discomfort to others. At the same time, stereotypes of people in other social groups, as well as the connection between them and their own social group members, influence their own perceptions and judgments (20). Based on this, this research inferred that the supporters of an over-moral sense tend to expect others to meet a higher moral standard. Therefore, we assumed that excessive moral sense could be another cause of cyberbullying, and so it was taken as one of the antecedent variables. Further, in this research, excessive moral sense is defined as the use of unreasonable moral standards with no personal boundary regarding others’ behaviors.

### 1.3 Cyberbullying perceived value

What is more, social cognitive research has the primary aim of investigating the interactive process, through studies on the influence of individuals, behaviors, and environments (27). Strohmeier and Gradinger (8) pointed out that cyberbullying is a kind of meta-process. That is to say, it is related to the influence on self-perception of the participants in the event, the participants themselves, their behaviors, and the environment. According to previous studies, cyberbullying is related to some cognitive responses and psychological influences (28). Therefore, this research examined the influence of cyberbullying behavior on the cognitive and psychological factors of the perpetrators, as well as the forming mechanism of cyberbullying among perpetrators in this social process.

Cyberbullying is not a one-time-only behavior, which is similar to traditional bullying. It is thus important to understand the continued cyberbullying intention of the perpetrators. Therefore, this research investigated the influence of cognitive and psychological factors on continued cyberbullying intention.

This will help us better understand the reason related to continued cyberbullying intention. Among the social factors, perceived value is seen as standards, rules, regulations, criteria, norms, or ideals, which serve as the foundation for any type of prioritized judgment (29). Perceived value is a cognitive weighing up of benefits and losses; the difference can be used to distinguish the different types of value perception. However, it can be further used to distinguish among various values rooted in various areas (30). Meanwhile, value refers to the comments from people after their interaction with objects and events (31). Therefore, the behavior of perpetrators on the Internet needs to be in accordance with their own moral standards. When everything perceived by perpetrators meets their standards, they will perceive a sense of value accordingly. This research, therefore, took perceived value as an outcome variable in the hypothetical model. Further, in this research, perceived value is defined as the feeling that helps people living in the world of the Internet to establish meaning.

### 1.4 Subjective happiness

Happiness is the psychological perception sought after by people. In East Asian cultures, happiness is conceptualized as being associated with interpersonal relations. People living in East Asian cultures tend to be motivated to strike a balance between positive and negative influences, and happiness is better predicted by the perceived integration of oneself into social relations (32). Therefore, when perpetrators believe that the social relations in the world of the Internet meet their expectations, they will gain happiness. According to research, measures of individual well-being can only be reliably and empirically reflected based on experiences, and the reported subjective happiness is a wider concept than the traditional utilities of decision making (33). According to Maslow, living a better life is largely decided by the degree to which one’s needs are met. The more needs that are met, the happier people will become (34). Therefore, when perpetrators believe that they are in a superior position in the online world, and can judge the right or wrong of others, they could feel that they dominate the social relations online, and thus experience a positive feeling. In this research, happiness is defined as an outcome variable in the hypothetical model. Victims of cyberbullying live in an environment in which they feel attacked for a long time, which has negative effects on their physical and mental health.

### 1.5 Continued cyberbullying intention

Viewed from a theoretical perspective, bullying can be seen as occurring on a continuum (15), where a consistent cyberbullying experience (of being bullied) could continue to strengthen these thoughts, and ultimately form and automatize a positive attitude towards cyberbullying (35). Based on this, cyberbullying could not be a one-time behavior, but rather is repetitive, with a periodic feature or intensity. Of course, it is an event that people are not willing to see. Moreover, continuity is one of the forms of behavior after being chosen (31). Therefore, after a perpetrator exhibits cyberbullying behavior, they are likely to exhibit the same behaviors again later. Therefore, this research set continued cyberbullying intention as an outcome variable. In this research, continued cyberbullying intention is defined as the perpetrator’s intention to continue cyberbullying others.

### 1.6 Current study

In a nutshell, cyberbullying is a serious problem that exists in this digital era. In order to solve this problem, we must have a clear picture of cyberbullying (36). Developmental theory can help to explain the reasons behind these events, and can provide opportunities to upgrade the intervention measures (37). That is to say, understanding the most possible behaviors and relevant variables leading to cyberbullying can provide potential solutions (4). Therefore, based on the SCT framework, this research aimed to construct and verify the Continued Cyberbullying Model (cause-effect model) related to the cyberbullying behavior of perpetrators.

## 2 Theoretical framework and research hypotheses

### 2.1 Theoretical background

Social Cognitive Theory (SCT) is one of the paradigms in psychological studies which interprets human behavior as the function of social cognitive influence in three major categories (i.e., human, behavior and environment) (38). At the same time, social cognitive theory is one of the major explanations of the Motivation Theory, which assumes that an internal process can lead to behavioral results (27). Therefore, SCT is a widely-accepted theory to interpret individual behaviors (39). From the SCT perspective, human behavior can be understood by observing psychological factors; individual behavior and the upcoming behaviors can be influenced by the shaping of the environment. Therefore, SCT has taken psychological factors as the auxiliary factors which influence human behaviors and interactions (38).

It is considered that bullying is increasingly influenced by social cognition which is relevant to social status and popularity (37). Therefore, SCT is an important theoretical framework for understanding the complexity of bullying behavior and the social characteristics of the engagement of bullying. In this way, bullying is considered as an issue of social relations. The interaction between individuals and their social environments supports this kind of conceptualization (40). Therefore, this research utilized SCT as its theoretical framework to interpret the cause of cyberbullying behaviors and continued cyberbullying intention.

### 2.2 Research hypotheses

#### 2.2.1 Relation between over-self-perception and cyberbullying behaviors

This has made overconfidence a common focus in socio-psychological research (23), as it is one of the most influential factors among the mistakes and biases of people in the judgments of themselves as well as their social judgments, and it has the highest potential cost (41). According to previous research, overconfidence can cause some severe consequences. For example, people tend to overvalue their own performance, and undervalue that of others, and then believe that they are better than other people (21). What is more, overconfident people can sometimes maintain their own influence and status in other people’s eyes, although they have the experience of making imprecise judgments (42). Moreover, according to the research, personal experiences make people focus extra attention on some commonly-seen events (21). Based on this, we can infer that overconfident people can be over-critical in terms of moral standards.

What is more, based on the perspective of SCT, people’s opinions can influence their behaviors and environment, while behaviors can change their thoughts and environments, and the environment can influence the thoughts and behaviors of individuals (27). Therefore, overconfident people could have behavioral biases, leading to the adoption of wrong standards as moral requirements of others. Cyberbullying is thus more likely to be exhibited by overconfident individuals.

Although related criminological theories posit that morality can curb crimes, in particular circumstances, morality can also result in crimes (43). Previous studies have pointed out that in the developmental process of moral self, individuals construct a right or wrong standard which serves as a guideline for their actions (44).

Excessive moral sense belongs to a type of moral coercion and hijacking of morality. We can also assume that people with an excessive moral sense are more likely to exhibit cyberbullying behaviors. Based on the above-mentioned results in the literature, we proposed the following hypotheses:

*H1*: Overconfidence can positively predict cyberbullying behaviors.

*H2*: Overconfidence can positively predict excessive moral sense.

*H3*: Excessive moral sense can positively predict cyberbullying behaviors.

#### 2.2.2 Relations between cyberbullying behaviors and perceived value & sense of happiness

People around the world are increasingly immersed in the world of the Internet, which extends beyond time, distance, location and national borders (44). The world of the Internet has become a cross-cultural community with people in different social groups. Netizens on the one hand engage in social activities, and establish their own values which they may impose on others. While SCT can help to interpret personal development, adaptation, and changes of people in various cultural environments (44), it assumes that people’s behavior can reflect their values (45). Therefore, it can also help us to interpret the cyberbullying behaviors of perpetrators and the shaped results of the perceived value. This is also in line with the core concept in SCT -- seeking a sense of agency, or they believe that they are able to have a significant influence on the major events in their lives (27). In other words, when individuals are constructing standards, they could possibly reconstruct the negative behaviors and cognitions into positive or valuable behaviors (44).

For example, previous research pointed out that a type of motivation is to gain happiness by harming others. Another group of teenagers may not really care about the harm to the target (46). Based on teasing, they believe that they can please peers, build connections and gain acknowledgement, then enjoy a higher social status (47). What is more, as SCT points out, motivation is largely determined by the expected positive results of taking real actions (27). However, the research of Varjas et al. (46) found that the reason behind cyberbullying largely comes from the frequency of internal motivation. That is to say, when people take certain actions, they have already considered the possible influence, and then they act, while perpetrators with a lower degree of self-control are less sympathetic. Therefore, they are less likely to enjoy the benefits of power over the victims, while at the same time, they are less likely to realize the pain that they cause. This could make perpetrators gain a certain degree of pleasure from cyberbullying others. Therefore, after perpetrators have shown cyberbullying behaviors, they could have a twisted sense of subjective happiness. Based on the above-mentioned results reported in the literature, we proposed the following hypotheses:

*H4*: Cyberbullying can positively predict perceived value.

*H5*: Cyberbullying can positively predict subjective happiness.

#### 2.2.3 Relations between perceived value, sense of happiness and continued cyberbullying intention

According to SCT, behavioral decisions are the result of interactions between individuals and environments. Individual factors reflect the internal factors such as individual knowledge, experience, attitude, and mental status; environmental factors are the external factors shaping the interaction between individuals and the environment (48), while continuous use behavior is the result of the joint interaction of human, environment and behavior (49). In other words, the continuous cyberbullying behavioral intention of the perpetrators is influenced, and the influence comes from the perceived changes of lives (environment) resulting from their behaviors. When adolescents feel their values are controlled, they attempt to restore them through aggression by disparaging others in front of an audience. Adolescents criticize the values they reject and aspects they do not identify with, thereby rationalizing the attack (47).

Zhou (25, 26) found that self-perception has an influence on continuity of people’s behavior. On shopping platforms (50), online social games (31), social media (51), government service systems (52) and in other various research scenarios, it has been found that perceived value has a positive influence on continued cyberbullying intention. What is more, in causal activities (53), virtual trips (54), overseas studies (55) and other various research scenarios, it has been found that the sense of happiness has a positive influence on continued cyberbullying intention. Overall, with a positive perception, people will have the intention to take actions one more time. Based on the abovementioned results reported in the literature, we proposed the following hypotheses:

*H6*: Perceived value can positively predict continued cyberbullying intention.

*H7*: Sense of happiness can positively predict cyberbullying behaviors.

### 2.3 Research model

**Figure 1:**
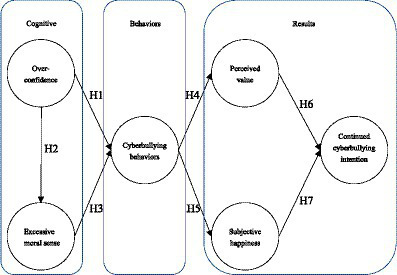
The hypothesis model.

Based on the perspective of SCT, young people who act as perpetrators of cyberbullying believe that they can receive certain rewards (e.g., enhanced social status, access to resources) from their behavior (40). That is to say, when students are more confident about the increase in interest in their behaviors, they are more likely to perform these behaviors. Based on this concept, we constructed a research model of continued cyberbullying intention with six variables. There are seven hypotheses to understand the relation among these variables, as illustrated in Figure 1.

## 3 Methods

### 3.1 Procedure

This study was investigative research based on a cross-sectional design. It adopted snowball sampling using *Wenjuanxing* (one of the most well-known platforms for surveys in China) to disseminate questionnaires online.

There are emerging cyberbullying issues related to student netizens. The previous research placed more emphasis on teenagers, rather than on university students (56). Therefore, the research subjects in this study were university students in China. The research was approved by the Faculty of Psychology of Beijing Normal University (approval number: 202202240017). The research adopted snowball sampling, and teachers assisted us in posting the recruitment notice and online questionnaires to students, and later the participants were asked to forward the questionnaires on to other students. There was no reward given to participants after responding to the questions. Moreover, in order to receive a more accurate response, the title of this questionnaire was set as “Internet using behavior” rather than “cyberbullying.” The research subjects were enrolled adult university students, with the habit of using social media. Data collection began on May 15, 2022 and continued until a sample of 1,200 questionnaires was collected; the link to the questionnaire was then closed.

On the first page of the questionnaire, the research objectives, rights and interests of participants, and the anonymous nature of the data collection were introduced. Participants were also asked to fill out the questionnaire in an online commenting background.

The questionnaire was fully anonymous; the names and contact information of participants were not collected. Participants were told that their involvement in this research was totally voluntary, and that they could exit the questionnaire at any time without any negative consequences. They were also informed that once they started to answer the questions, it was equivalent to filling in an informed consent form.

### 3.2 Participants

Our sample can be considered as large, as normally the minimal sample for this type of study is 200. The number of participants in this research was thus far above the requirement. A total of 1,200 participants completed the questionnaire, but after exclusion of 152 incomplete questionnaires and those with limited (too short) answering time, there were 1,048 effective participants, giving an effective recovery rate of 87.3%. All participants were adult students (18 years of age and over). The average age of participants was 20.29, with a standard deviation of 1.43 years of age. Detailed participant information is shown in Table 1.

**Table 1:** Descriptive statistics.

Variables	Details
1. Gender	Male: 465 (44.4%) Female: 583 (55.6%)
2. Grade	Freshman: 487 (46.5%) Sophomore: 188 (17.9%) Junior: 313 (29.9%) Senior: 60 (5.7%)
3. Days spent on social media per week	1–3 days: 6 (0.5%) 4–6 days: 67 (6.4%) 7 days: 975 (93.1%)
4. Time spent using social media each time it is accessed	Less than 1 h: 167 (15.9%) 1–3 h(s): 604 (57.7%) 3–5 h: 256 (24.4%) More than 5 h: 21 (2%)
5. Most frequently used social media (Multiple choice, 5 maximum)	Weibo: 1021 (19.5%) Little Red Book: 949 (18.1%) Bilibili: 856 (16.3%) Zhihu: 545 (10.4%) TikTok: 922 (17.6%) KuaiShou: 451 (8.6%) Volcane Video: 334 (6.4%) Others: 162 (3.1%)
6. Did you ever receive malicious comments on the Internet?	Yes: 623 (59.4%) No: 425 (40.6%)
7. Did you ever leave malicious comments on the Internet?	Yes: 793 (75.7%) No: 255 (24.3%)

### 3.3 Measurement

This was quantitative, confirmatory research, which used a questionnaire survey to collect data. The questionnaire in this research was divided into two parts, newly developed items and adapted items. Items for the constructs of overconfidence, excessive moral sense, and cyberbullying were designed based on the definition of the variables, whereas the items for perceived value, happiness and continued cyberbullying intention were designed with reference to previous research tools. Items were back translated and adapted based on the current context. The questionnaire was reviewed by three experts in the field of education in order to check the completeness, wording comprehensibility, and so on to ensure expert validity. The questionnaire utilized a 5-point Likert Scale (from 1 to 5 representing *Strongly Disagree* to *Strongly Agree*, respectively). The six scales are described as follows.

#### 3.3.1 Overconfidence

Overconfidence refers to the inconsistency between people’s perceived self-confidence (which is higher) and the real situation. Six items were designed for this construct in order to measure whether participants were overconfident and irrational. Example items include: “Most of the time, I think my opinions are correct” and “Even if someone corrects my thoughts, I still believe that mine are right.” We used a 1 to 5 rating scale, where the higher the score, the higher level of over-confidence the participant reported.

#### 3.3.2 Over-moral sense

Excessive moral sense is a type of moral coercion and the hijacking of morality; it refers to irrational or unlimited moral requirements regarding others’ behaviors. Six items were designed for this construct in order to measure whether participants were overconfident and had moral standards that were too high or irrational. Example items are: “I cannot accept a single mistake made by others” and “When other people make mistakes, I would blame them immediately.” We used a 1 to 5 rating scale, where the higher the score, the higher the level of excessive moral sense the participant reported.

#### 3.3.3 Cyberbullying behaviors

Cyberbullying behaviors refer to people acting as perpetrators by making malicious attacks on others on the Internet. We explained to participants that these questions were used to check personal continued behavioral intentions according to events that will happen in the future. With reference to the Cyberbullying Experience Scale by Doane et al. (57), revisions were made, and eight items were used to measure whether participants had cyberbullying behaviors. Example items include: “I once left malicious comments about others on the Internet” and “I once mocked others online publicly.” According to the research of Doane et al. (57), the Cronbach’s α value of this variable lies between 0.84 and 0.97. We used a 1 to 5 rating scale, where the higher the score, the more cyberbullying behaviors the participant reported.

#### 3.3.4 Perceived value

Perceived value means the feeling that helps people living in the world of the Internet to establish meaning. Based on the above-mentioned concepts, this research referred to the questionnaire of Kern et al. (58), and made revisions to the items. Six items were used to measure whether life in the world of the Internet provides a higher sense of value to their lives. Example items include: “Being active in the world of the Internet gives my life a higher sense of value” and “Being active in the world of the Internet makes my life gain a higher sense of belonging.” According to the research of Kern et al. (58), the Cronbach’s α value of this variable is 0.85. We used a 1 to 5 rating scale, where the higher the score, the higher perceived value the participant reported.

#### 3.3.5 Subjective happiness

Subjective happiness refers to the feeling that helps people living in the world of the Internet to have positive feelings. This research made reference to the Sense of Happiness Scale of Ye et al. (59), with revisions made. Nine items were used to measure whether life in the world of the Internet provides more positive perceptions to the life of participants. Example items include: “In the world of the Internet, I am much happier” and “In the world of the Internet, I am more pleased and excited.” According to the research of Ye et al. (59), the Cronbach’s α value of this variable is 0.96. We used a 1 to 5 rating scale, where the higher the score, the higher level of happiness the participant expressed.

#### 3.3.6 Continued cyberbullying intention

Continued Cyberbullying Intention refers to the intention to continue cyberbullying others. A construct with six items was designed to measure whether participants had the intention to continue cyberbullying others. Before answering these six questions, a statement was presented to the participants: “The following questions require your likely behavioral intention when facing situations of this kind in the future.” Example items include: “When I see something online that I’m not satisfied with, I would like to leave some aggressive comments” and “When I see something online that I’m not satisfied with, I would ask friends to leave some aggressive comments together with me.” We used a 1 to 5 scale rating, where the higher the score, the higher intention the participant had to continue their cyberbullying behavior.

## 4 Results and discussion

This study was confirmatory research which used SPSS 23.0 and AMOS 20.0 for the statistical analysis. First-order confirmatory analysis, reliability and validity analysis, and model fit analysis were performed in order to confirm that both the constructs and items could reach the suggested standards, and finally to examine the research model.

### 4.1 First-order confirmatory factor analysis

Before testing a model, assessing the measurement model is necessary. Therefore, we adopted first-order confirmatory factor analysis. Statisticians suggest that the value of χ^2^/*df* should be lower than 5; the value of RMSEA should be lower than 0.10; the values of GFI and AGFI should be higher than 0.80; and items with factor loading (FL) values below 0.50 should be deleted from the original survey (60, 61), as demonstrated in Table 1. Based on the above-mentioned statistical criteria, items which did not meet the factor loading standard were deleted as follows: the number of items for overconfidence was reduced from six to four; excessive moral sense was reduced from six to four; cyberbullying was reduced from eight to six; perceived value was reduced from six to four; subjective happiness was reduced from nine to six; and continued cyberbullying intention was reduced from six to five.

This research utilized the measurement of the external validity of items to interpret the range (62). A *t* test was conducted on the top and bottom 27% of participants responding to each question in this survey; when the *t* value is higher than 3 (***p < 0.001), it is considered to reach the significance level in terms of external validity. As shown in Table 2, the *t* values of the constructs fell between 22.79 and 59.55 (***p < 0.001), which signifies that all question items in this research have external validity, and can be applied to various research scenarios (63).

**Table 2:** First-order confirmatory analysis.

Model fit	χ^2^	*df*	χ^2^/*df*	RMSEA	GFI	AGFI	FL	*t*
Cut-off value	–	–	< 5	< 0.10	> 0.80	> 0.80	> 0.50	> 3
Overconfidence	0.6	2	0.3	0.01	0.99	0.99	0.65~0.77	22.79~28.26
Excessive moral sense	0.3	2	0.15	0.01	0.99	0.99	0.73~0.89	29.00~35.90
Cyberbullying	16.6	9	10.84	0.03	0.99	0.99	0.81~0.89	41.21~55.17
Perceived value	20.6	2	10.3	0.02	0.99	0.99	0.75~0.95	40.66~47.49
Subjective happiness	17.3	9	10.92	0.03	0.99	0.99	0.74~0.91	28.53~40.54
Continued cyberbullying intention	16.2	5	30.24	0.05	0.99	0.99	0.69~0.96	35.67~59.55

### 4.2 Skewness and kurtosis coefficients

The values for skewness and kurtosis between −2 and + 2 are considered acceptable in order to prove normal univariate distribution (64). The results of the analyzes in this study all met the criteria, as depicted in Table 3.

**Table 3:** Skewness and kurtosis coefficients.

Constructs	Skewness		Kurtosis	
	Coefficient	Error	Coefficient	Error
Overconfidence	0.187	0.076	1.550	0.151
Excessive moral sense	1.001	0.076	1.027	0.151
Cyberbullying	0.827	0.076	0.077	0.151
Perceived value	−0.183	0.076	−0.327	0.151
Subjective happiness	−0.109	0.076	0.636	0.151
Continued cyberbullying intention	0.756	0.076	−0.166	0.151

### 4.3 Harman’s one-factor test

First of all, the KMO and Bartlett sphericity tests were conducted, which showed that KMO = 0.952, χ^2^ = 28400.18, *df* = 406, *p* < 0.001. It means that the sample data can be used for factor analysis. Then, using principal component analysis and the varimax orthogonal rotation, six common factors were extracted based on the criterion of eigenvalues greater than 1, accounting for 78.49% of the total variance, which is greater than 50%. The explanatory power of these six factors for the total variation ranges between 2.94 and 39.26%.

### 4.4 Reliability and validity analysis

This research confirmed the scale’s internal consistency with Cronbach’s α, and double checked the reliability with composite reliability (CR). According to Hair et al. (60), the value of Cronbach’s α and the CR value should be higher than 0.70. In this research, the value of Cronbach’s α lies between 0.80 and 0.95, and the CR value lies between 0.76 and 0.95, which is in accordance with the suggestion, as is depicted in Table 4.

**Table 4:** Reliability and validity analysis.

Constructs	*M*	*SD*	α	FL	AVE	CR
	–	–	> 0.70	> 0.50	> 0.50	> 0.70
Overconfidence	2.92	0.69	0.80	0.71	0.51	0.76
Excessive moral sense	2.46	0.91	0.91	0.84	0.72	0.91
Cyberbullying	1.84	0.88	0.95	0.87	0.75	0.95
Perceived value	2.58	0.90	0.93	0.89	0.79	0.94
Subjective happiness	2.82	0.85	0.94	0.85	0.73	0.94
Continued cyberbullying intention	2.28	1.08	0.95	0.89	0.80	0.95

Convergent validity is assessed by factor loading (FL) and average variance extracted (AVE). According to Hair et al. (60), the FL value should be higher than 0.50. Items lower than this value should be deleted. Items retained in this research all met the recommended standards, as the FL values fell between 0.71 and 0.89, as shown in Table 2. Hair et al. (65) suggested that the AVE value should be higher than 0.50 to show that the construct had convergent validity. The AVE values in the present study fell between 0.51 and 0.80, as shown in Table 4.

Discriminant validity can help to judge whether the constructs are independent of each other in the research model. According to Awang (66), in terms of the AVE root value of every construct, when it is higher than the Pearson Correlation Coefficient (PCC) of other constructs, it means that the construct has discriminant validity. According to the analysis results, all constructs in this research had discriminant validity, as shown in Table 5.

**Table 5:** Discriminant validity analysis of constructs.

Constructs	1	2	3	4	5	6
1. Overconfidence	(0.84)					
2. Excessive moral sense	0.49	(0.92)				
3. Cyberbullying	0.32	0.40	(0.93)			
4. Perceived value	0.21	0.16	0.40	(0.94)		
5. Sense of subjective happiness	0.21	0.13	0.34	0.79	(0.92)	
6. Continued cyberbullying intention	0.14	0.18	0.46	0.53	0.58	(0.94)

The value on the diagonal is the AVE root value, and other values are the correlation coefficient values.

### 4.5 Model fit analysis

Before verifying the model fit, the goodness-of-fit should be confirmed. The suggested values of each fit index are as follows: the value of χ^2^/*df* should be lower than 5 (60), the value of RMSEA should be lower than 0.1, the values of GFI, AGFI, NFI, NNFI, CFI, IFI and RFI should be higher than 0.80 (67), while the values of PNFI and PGFI should be higher than 0.50 (60). The values of the fit statistics in this research are as follows: χ^2^ = 1831.6, *df* = 370, χ^2^/*df* = 4.95, RMSEA = 0.06, GFI = 0.90, AGFI = 0.89, NFI = 0.94, NNFI = 0.94, CFI = 0.95, IFI = 0.95, RFI = 0.93, PNFI = 0.85, and PGFI = 0.77.

### 4.6 Path analysis

**Figure 2:**
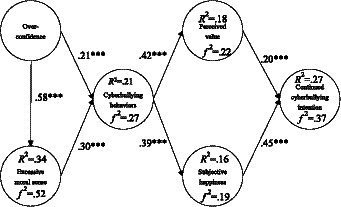
The results of the structural model; ***p < 0.001.

After checking the reliability and validity of the constructs, as well as the model fit, the next step is assessing the result of the structural model. According to the confirmatory test results: overconfidence had a positive influence on cyberbullying (*β* = 0.21***, *p* < 0.001), and a positive influence on excessive moral sense (*β* = 0.58***, *p* < 0.001); excessive moral sense had a positive influence on cyberbullying behaviors (*β* = 0.30***, *p* < 0.001); cyberbullying had a positive influence on perceived value (*β* = 0.42***, *p* < 0.001); cyberbullying behaviors had a positive influence on sense of happiness (*β* = 0.39***, *p* < 0.001); perceived value had a positive influence on continued cyberbullying intention (*β* = 0.20***, *p* < 0.001); and sense of happiness had a positive influence on continued cyberbullying intention (*β* = 0.45***, *p* < 0.001), as shown in Figure 2.

What is more, overconfidence had explanatory power of 34% for excessive moral sense, and the *f^2^* value was 0.52; overconfidence and excessive moral sense had explanatory power of 21% for cyberbullying behaviors, and the *f^2^* value was 0.27; cyberbullying behavior had explanatory power of 18% for perceived value, and the *f^2^* value was 0.22; cyberbullying behavior had explanatory power of 16% for sense of happiness, and the *f^2^* value was 0.19; perceived value and sense of happiness had explanatory power of 27% for continued cyberbullying intention, and the *f^2^* value was 0.37. These results are demonstrated in Figure 2.

### 4.7 Discussion

#### 4.7.1 Overconfidence and excessive moral sense have positive influences on cyberbullying behaviors

According to the results in this research, overconfidence and excessive moral sense have positive influences on cyberbullying behaviors. That is to say, when people have a higher degree of overconfidence, they are more likely to exhibit cyberbullying behaviors. When people have an overly high moral sense, it is also more likely to result in cyberbullying behaviors. What is more, according to this research, overconfidence has a positive influence on excessive moral sense. That is to say, when people have a higher degree of overconfidence, they are more likely to have overly high moral standards. These results are consistent with previous studies; for example, Moore and Healy (21) and Tenney et al. (42) pointed out that overconfidence can make people overestimate their own performance and further underestimate other people. Then they believe that they themselves are better than others, and tend to maintain their own influence and their status in other people’s eyes. Therefore, people with overconfidence, based on cognitive bias, are more likely to have biased behaviors. Sijtsema et al. (68) found that the perpetrators are considered popular at school, which is a sharp contrast to the victims. Moreover, according to Moore and Healy (21), personal experiences make people focus extra attention on some commonly-seen events. This is also in accordance with the opinion of Schunk and DiBendetto (27) who found that the thoughts of people can influence their behaviors and environment. Based on this, overconfident people can be over-critical in terms of moral standards, leading to the adoption of wrong standards as moral requirements of others. Moreover, many people criticize just for the sake of criticism; the matter itself has no right or wrong, but merely serves to prove one’s own righteousness. This is in accordance with what was mentioned by Marcum et al. (69) who found that perpetrators often feel justified, happy and proud of their actions. According to Silver and Silver (43), although related criminological theories posit that morality can curb crimes, in particular circumstances, morality can also lead to crimes. For example, some intuitive morality with binding force can result in group violence. This opinion is in accordance with Bandura (44) – in the developmental process of moral self, individuals construct the right or wrong standard, which serves as a guideline for their actions. According to Lee (70), in the digital world, users are less likely to be restricted by traditional values and social morality. They can express themselves with no restrictions, based on their own preferences, likes and dislikes, as well as values. Furthermore, Zhou (25, 26) expressed that people with an excessive moral sense focus on socially hotly debated events and sensitive issues. The online hypocritical commenters abuse the function of moral judgment, and narrowly overemphasize the exclusivity and absolutism of moral judgment, even replacing legal judgment with moral judgment. In this case, overconfidence and excessive moral sense (twisted justice) become the major causes of cyberbullying events. This is a better interpretation of the large number of cyberbullying behaviors, but perpetrators may have no awareness of the reason for their behaviors.

#### 4.7.2 Cyberbullying has a positive influence on perceived value and sense of happiness

According to this research, cyberbullying has a positive influence on perceived value and sense of happiness. That is to say, the more times perpetrators exhibit cyberbullying behaviors, the more value and happiness what they do in the world of the Internet will provide them with. This is also in accordance with the finding of Varjas et al. (46), namely that one of the motivations is gaining happiness by harming others. Another is purely pleasing. Perpetrators may not really care whether the victims are harmed or not. Moretti & Herkovits (47) found that perpetrators, through mockery, believe they can entertain their peers and establish connections. Aggression is learned and rooted in their social skills by seeking the recognition and relationships of peers and adversaries, thus granting them social status. Furthermore, this type of twisted and unrighteous value construction and formation of a sense of happiness can be interpreted from subsequent research. According to Bandura (45), SCT assumes that people’s behaviors can reflect their own values. In the world of the Internet with various social groups and its cross-cultural nature, people engage in online social activities based on this, and establish their own values. However, Schunk and DiBenedetto (27) pointed out that motivated behaviors largely depend on the expected results of real action. Therefore, people seek a sense of agency, or believe that they can exert a greater influence on the major events in their lives. This also leads to the cognitive reconstruction of negative behaviors into behaviors with positivity or with value, in the process of constructing personal standards. The above-mentioned literature can help to explain the cyberbullying behaviors of the perpetrators, as well as the shaped results of the online perceived value.

Although cyberbullying behaviors have a positive influence on their sense of happiness, Kim et al. (1) believe that perpetrators are not likely to realize the pain suffered by victims in the process of bullying. They have a feeling of pleasure during the cyberbullying process. This is in line with the opinion in SCT that people’s motivation largely depends on the expected result after taking actions (27). However, Navarro et al. (71), along with Giumetti and Kowalski (4), believe that perpetrators are more likely to suffer worse mental health results which have a negative influence on their life satisfaction or happiness. However, the confirmatory results of this research are not in line with this opinion. Although this result is hard to interpret, from the theory that happiness is relative, gaining happiness is dependent on meeting one’s needs (72). The results can be explained by Nixon (73) who found that there is relativity between participation in cyberbullying and emotional disorder. This makes the explanation easier to understand. This is also in accordance with Telic theories – happiness can be experienced after a certain status is reached, certain goals are realized, or certain needs are met. That is to say, when they believe that doing something is right, and their goal is reached, happiness will be gained. However, the negative sense of gain needs to be reversed via attention and coaching from parents, professors and teachers. The perpetrators will need to realize that it is not a positive cognition and emotional experience to acquire value and a sense of happiness from the pain of others, but is rather a negative experience.

#### 4.7.3 Perceived value and sense of happiness have a positive influence on continued cyberbullying intention

According to the results of this research, perceived value and sense of happiness have positive influences on continued cyberbullying intention. That is to say, when perpetrators have a higher sense of value and happiness, they are more likely to continue to bully others. This result is also in accordance with Varjas et al. (46): one of the reasons that perpetrators cyberbully others is to make themselves feel better. What is more, Zhou (25, 26) believes that self-perception has an influence on people’s consistency. Moreover, Chang (31), Wang et al. (52), Zhao et al. (51), and Zhu et al. (50) all confirmed that perceived value has a positive influence on continued cyberbullying intention. What is more, Han (53), Kim et al. (54), and Jamaludin et al. (55) found that the sense of happiness has a positive influence on continued cyberbullying intention. Moreover, according to the research by Festl and Quandt (74), bullying is a stable behavior, as most perpetrators have shown this behavior via the Internet. These results are in accordance with Huo and Li (49); based on SCT instruction, consistent use behavior is the result of mutual interaction among people, environment and behavior. Overall, with a biased positive perception, people will have the intention to take actions one more time.

### 4.8 Research limitations and future studies

There are limitations to this research that should be noted. This study aimed to discover the causes of cyberbullying behaviors and the continued cyberbullying intention of perpetrators; therefore, the opinions of other interested groups were not studied. However, Macaulay et al. (17) pointed out that participants in cyberbullying can be generally categorized into three groups: abettors (perpetrators), targets in humiliation (victims), and observers of the event (bystanders). However, in this research, targeting the perpetrators makes it impossible to understand the opinions and thoughts of all parties of the cyberbullying event. Therefore, in the coming research, online ethnography, interviews and other qualitative research methods can be used to further discuss the influence of cyberbullying behaviors.

What is more, a significant conclusion of this research is that when perpetrators believe what they are doing (i.e., cyberbullying) is valuable or that they can gain positive feelings from it, they will continue to exhibit cyberbullying behaviors; this requires our attention. As there are few studies with such a result, it is difficult to determine further explanations; therefore, more research is needed to build a detailed mechanism related to the causes. Many cases in the real world indicate that perpetrators are satisfied with their punishment of others, and therefore they exhibit continued cyberbullying behavior.

It should be noted that the fundamental reason for cyberbullying could change along with the aging process (37). However, as this research was based on a cross-sectional design, it was not possible to understand whether the cause of cyberbullying by perpetrators could change over time. Therefore, it is suggested that in the coming studies, a longitudinal design can be adopted, and the influence of perpetrators, victims, and bystanders in an environment with long-term cyberbullying can be examined, as well as whether there is any change in terms of the cause of cyberbullying. Certainly, as students grow older, education in cyberbullying prevention can adopt various approaches and foci. The coming research can also aim to understand the influence of cyberbullying prevention education on students, as well as the influence of educational policies and goals. For example, China stresses the importance of civic education (including the theoretical, political, and practical aspects of citizenship), moral cultivation, and so on, in order to raise students’ moral awareness. These policies are worth further research endeavors and discussion. As the sample collected from young people is quite limited demographically and statistically, it could lead to prevalent problems (75). Bullying factors may vary across different age groups, and the educational stages and contexts also differ. Therefore, the following research can investigate other cyberbullying factors based on different context, and then develop theory models accordingly. In this case, better intervention and prevention measures can be put into practice, which can then reduce or prevent potential cyberbullying.

## 5 Conclusions and suggestions

### 5.1 Conclusion

Based on SCT, this research formed a consistent cyberbullying intention research model with six variables, based on the use of seven hypotheses to understand the relationship among the variables. The research results are as follows: 1. Overconfidence and excessive moral sense have positive influences on cyberbullying behaviors; 2. Overconfidence has a positive influence on excessive moral sense; 3. Cyberbullying behaviors have a positive influence on perceived value and sense of happiness; and 4. perceived value and sense of happiness have positive influences on continued cyberbullying intention.

Overall, this research confirms that overconfidence and sense of happiness are significant external variables with an influence on cyberbullying, which is in line with the public opinion that cyberbullying is based on the misperception of perpetrators. What is more, this research found that cyberbullying provides perceived value and a sense of happiness to perpetrators, which should be seen as a twisted cognitive experience, as this makes perpetrators have wrong thoughts of hoping to continue this type of malicious cyberbullying behavior. This type of result could lead to perpetrators’ unawareness of their own cyberbullying behaviors, instead believing that they are upholding justice and maintaining moral order.

### 5.2 Contributions

This research makes three contributions to the literature. First of all, based on the self-serving bias, this research proposes a new variable called excessive moral sense, which is a term that has been rarely discussed in academia. However, in daily life, using unreasonable moral standards to force others to follow rules is common, and leads to moral hijacking. Therefore, this variable is helpful for people to gain insights into moral and cognitive psychology. Secondly, this research has proposed a behavioral pathway model to interpret the forming mechanism of cognition-behavior-result, and to further understand the possible factors of continued cyberbullying behavior. Certainly, this pathway model can also be used to interpret the forming mechanism of other negative behaviors. Last but not least, most research cases in cyberbullying start from the perspective of victims, and to explore the way to help them get rid of harm and pain. However, understanding the underlying reasons for the perpetrators’ behaviors also has great significance. The results of the analysis in this research will be helpful for parents, teachers, schools and government bodies to better plan and implement measures to stop cyberbullying behaviors after understanding the reasons for self-serving bias.

### 5.3 Suggestions

Due to the popularity of Internet technology, people have more time online to communicate and exchange ideas. However, this has also made cyberbullying more frequent, and it has a greater influence on every age group and every social group than before. This has also made cyberbullying an urgent and significant public health issue. Therefore, it is important to propose more interventions and preventive measures. Based on our research results, these factors are quite related to the real-life situation. As it is hard to curb cyberbullying behaviors, government bodies need to roll out laws and regulations, as well as policy advocations. Education authorities should establish preventive and intervention systems based on schools and families, as well as establishing school-family cooperation systems. This would allow perpetrators of the cyberbullying events to realize that the Internet is not a lawless land, and would allow victims to know the ways to seek help, while also allowing by-standing observers to know the ways of providing help, rather than being additional perpetrators.

At the same time, empathy and putting oneself in others’ shoes is significant. With better empathy, people will consider more the harm to others. What is more, as one of the most important Chinese social media platforms, Weibo, WeChat, Douyin (Tiktok), Xiaohongshu and Zhihu can be used as a major channel to launch legal and moral education activities. Furthermore, excessive moral sense can be seen as a type of moral blackmail, with no moral cap. Therefore, schools with moral education should allow students to understand the correct moral standard, and help them to have respect, sympathy, and empathy, and learn to use polite and appropriate approaches to communicate and express themselves. This would allow them to use appropriate standards for self-discipline when viewing the comments and behaviors of others. Apart from education in morality and cyberbullying prevention, the Internet supervision departments in government should promulgate related laws and regulations, punishments and supervision policies, which can prevent the Internet from being the home to cyberbullying, and can effectively prevent cyberbullying behaviors. A legal basis for punishment and comprehension would serve as one of the protections for victims.

## Data availability statement

The raw data supporting the conclusions of this article will be made available by the authors, without undue reservation.

## Ethics statement

The studies involving humans were approved by ethics committee at the Faculty of Psychology, Beijing Normal University. The studies were conducted in accordance with the local legislation and institutional requirements. The participants provided their written informed consent to participate in this study. Written informed consent was obtained from the individual (s) for the publication of any potentially identifiable images or data included in this article.

## Author contributions

J-HY: Writing – review & editing, Writing – original draft, Resources, Methodology, Formal analysis, Conceptualization. XY: Writing – review & editing, Writing – original draft, Supervision, Conceptualization. WN: Writing – review & editing. MW: Writing – review & editing. Y-SL: Writing – review & editing.
